# Supplementary figures and images for: Fluctuations in coral reef fish densities after environmental disturbances on the northern Great Barrier Reef
Source: PeerJ. 2019 Apr 8;7:e6720. doi: 10.7717/peerj.6720 (PMC6459176; doi:10.7717/peerj.6720)

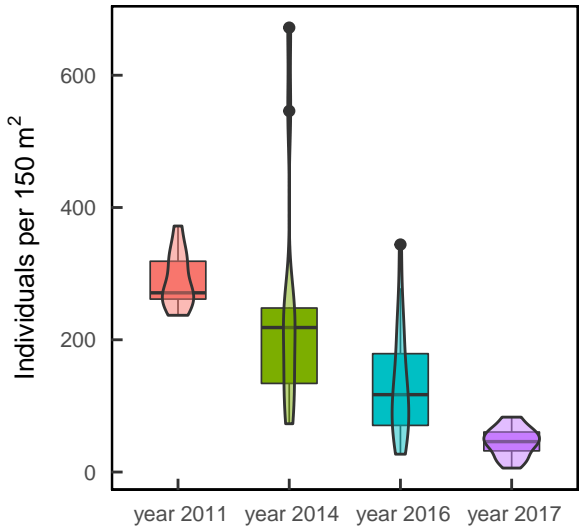

Supplement: Supplemental Information 1 — Beanplots and boxplots are displaying median and interquartile of fish abundance. [file peerj-07-6720-s001.pdf]

Individuals per 150 m<sup>2</sup>

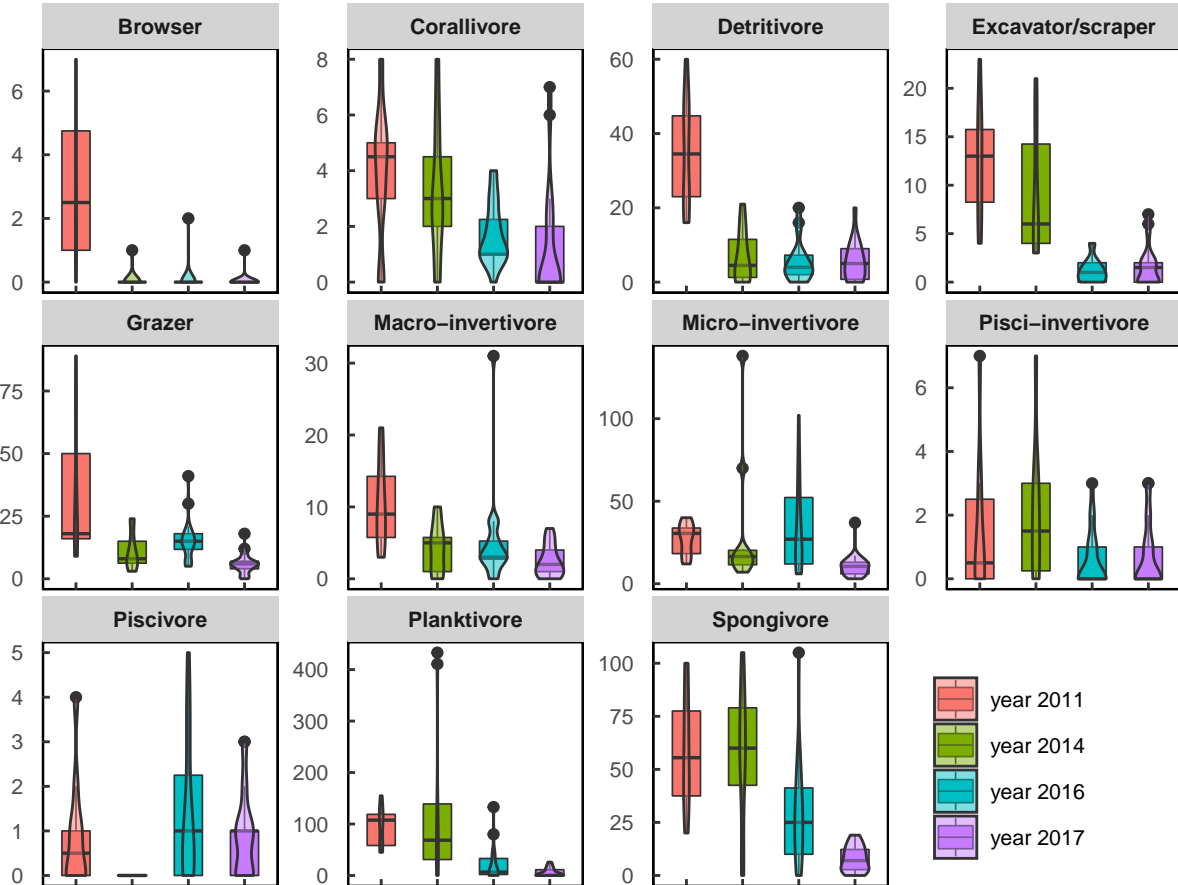

Supplement: Supplemental Information 2 — Beanplots and boxplots are displaying median and interquartile of fish abundance (A) per Site 1 or Mermaid Cove and (B) per Site 2 or Northern Horseshoe. Note that due to the high variation in fish abundance per functional groups, the y-axes are not similar. [file peerj-07-6720-s002.pdf]
