# Supplementary material for: Fluctuations in coral reef fish densities after environmental disturbances on the northern Great Barrier Reef
Source: PeerJ. 2019 Apr 8;7:e6720. doi: 10.7717/peerj.6720 (PMC6459176; doi:10.7717/peerj.6720)
Supplement: Supplemental Information 3 — The fish species classification followed Wernberg et al. (2013) and MacNeil et al. (2015). [file peerj-07-6720-s003.docx]

| genus_and_species | functional.group | species.size |
| --- | --- | --- |
| Abudefduf bengalensis | Planktivore | Large |
| Abudefduf sexfasciatus | Planktivore | Large |
| Acanthochromis polyacanthus | Planktivore | Large |
| Acanthurus auranticavus | Grazer | Large |
| Acanthurus blochii | Grazer | Large |
| Acanthurus grammoptilus | Grazer | Large |
| Acanthurus nigricauda | Grazer | Large |
| Acanthurus nigrofuscus | Grazer | Large |
| Acanthurus olivaceus | Detritivore | Large |
| Acanthurus thompsoni | Planktivore | Large |
| Amblyglyphidodon curacao | Planktivore | Large |
| Amblygobius phalaena | Detritivore | Large |
| Amphiprion akindynos | Planktivore | Small |
| Apogon apogonides | Planktivore | Large |
| Arothron nigropunctatus | Macro-invertivore | Large |
| Aspidontus taeniatus | Micro-invertivore | Large |
| Balistapus undulatus | Macro-invertivore | Large |
| Balistoides undulatus | Macro-invertivore | Large |
| Balistoides viridescens | Macro-invertivore | Large |
| Caesio teres | Planktivore | Large |
| Caranx melampygus | Piscivore | Large |
| Cephalopholis boenak | Pisci-invertivore | Large |
| Cephalopholis cyanostigma | Pisci-invertivore | Large |
| Cephalopholis sp | Pisci-invertivore | Large |
| Chaetodon aureofasciatus | Corallivore | Large |
| Chaetodon auriga | Micro-invertivore | Large |
| Chaetodon baronessa | Corallivore | Large |
| Chaetodon citrinellus | Micro-invertivore | Large |
| Chaetodon ephippium | Micro-invertivore | Large |
| Chaetodon kleinii | Micro-invertivore | Large |
| Chaetodon lineolatus | Micro-invertivore | Large |
| Chaetodon melannotus | Corallivore | Large |
| Chaetodon ocellicaudus | Corallivore | Large |
| Chaetodon plebeius | Corallivore | Large |
| Chaetodon plebius | Corallivore | Large |
| Chaetodon rainfordii | Corallivore | Large |
| Chaetodon trifascialis | Corallivore | Large |
| Chaetodon trifasciatus | Corallivore | Large |
| Chaetodon ulietensis | Micro-invertivore | Large |
| Chaetodon unimaculatus | Corallivore | Large |
| Chaetodon vagabundus | Micro-invertivore | Large |
| Cheilinus chlorourus | Macro-invertivore | Large |
| Cheilinus fasciatus | Macro-invertivore | Large |
| Cheilinus trilobatus | Macro-invertivore | Large |
| Cheilinus undulatus | Macro-invertivore | Large |
| Cheilodipterus quinquelineatus | Planktivore | Large |
| Cheilodipterus sp. | Planktivore | Small |
| Cheiloprion labiatus | Planktivore | Small |
| Chelmon rostratus | Micro-invertivore | Large |
| Chlorurus microrhinos | Excavator/scraper | Large |
| Chlorurus sordidus | Excavator/scraper | Large |
| Chlorurus sordidus | Excavator/scraper | Large |
| Choerodon monostigma | Pisci-invertivore | Large |
| Choerodon schoenleinii | NA | Large |
| Chromis viridis | Planktivore | Small |
| Chrysiptera cyanea | Grazer | Small |
| Chrysiptera rex | Grazer | Small |
| Chrysiptera rollandi | Micro-invertivore | Small |
| Cirripectes sp. | Detritivore | Large |
| Coris batuensis | Micro-invertivore | Large |
| Coris caudimacula | Micro-invertivore | Large |
| Coris gaimard | Macro-invertivore | Large |
| Cromileptes altivelis | Piscivore | Large |
| Ctenochaetus striatus | Detritivore | Large |
| Ctenochaetus binotatus | Detritivore | Large |
| Ctenochaetus striatus | Detritivore | Large |
| Dascyllus aruanus | Planktivore | Small |
| Dascyllus reticulatus | Planktivore | Small |
| Dischistodus melanotus | Detritivore | Large |
| Dischistodus perspicillatus | Detritivore | Large |
| Dischistodus prosopotaenia | Detritivore | Large |
| Dischistodus pseudochrysopoecilus | Detritivore | Large |
| Ephinephelus maculatus | Piscivore | Large |
| Ephinephelus merra | Piscivore | Large |
| Epibulus insidiator | Pisci-invertivore | Large |
| Gomphosus varius | Micro-invertivore | Large |
| Halichoeres chloropterus | NA | Large |
| Halichoeres melanurus | Micro-invertivore | Large |
| Halichoeres nebulosus | Micro-invertivore | Large |
| Halichoeres sp. | Micro-invertivore | Large |
| Hemigymnus fasciatus | Macro-invertivore | Large |
| Hemigymnus melapterus | Macro-invertivore | Large |
| Hologymnosus annulatus | Pisci-invertivore | Large |
| Hologymnosus doliatus | Pisci-invertivore | Large |
| Labrichthys unilineatus | Corallivore | Large |
| Labroides dimidiatus | Micro-invertivore | Large |
| Lethrinus obsoletus | Pisci-invertivore | Large |
| Lethrinus olivaceus | Pisci-invertivore | Large |
| Lethrinus sp. | Pisci-invertivore | Large |
| Lutjanus bohar | Piscivore | Large |
| Lutjanus carponatus | Piscivore | Large |
| Lutjanus fulviflamma | Piscivore | Large |
| Lutjanus fulvus | Piscivore | Large |
| Lutjanus sp. | Piscivore | Large |
| Macolor niger | Pisci-invertivore | Large |
| Monotaxis grandoculis | Macro-invertivore | Large |
| Mulloidichthys flavolineatus | Macro-invertivore | Large |
| Naso brachycentron | Browser | Large |
| Naso tuberosus | Planktivore | Large |
| Naso unicornis | Browser | Large |
| Neoglyphidodon melas | Micro-invertivore | Large |
| Neoglyphidodon nigroris | Planktivore | Large |
| Neoniphon sammara | Pisci-invertivore | Large |
| Neopomacentrus azysron | Planktivore | Small |
| Novaculichthys taeniourus | Macro-invertivore | Large |
| Oxycheilinus digrammus | Pisci-invertivore | Large |
| Paraluteres prionurus | Micro-invertivore | Large |
| Parapercis sp | Piscivore | Large |
| Parupeneus barberinus | Macro-invertivore | Large |
| Parupeneus cyclostomus | Pisci-invertivore | Large |
| Parupeneus multifasciatus | Macro-invertivore | Large |
| Plagiotremus tapeinasoma | Micro-invertivore | Large |
| Plectorhinchus chaetodonoides | Micro-invertivore | Large |
| Plectroglyphidodon lacrymatus | Grazer | Small |
| Plectropomus | Piscivore | Large |
| Plectropomus pessuliferus | Piscivore | Large |
| Plectropomus sp. | Piscivore | Large |
| Pomacanthus sexstriatus | Spongivore | Large |
| Pomacentrus amboinensis | Spongivore | Small |
| Pomacentrus bankanensis | Spongivore | Small |
| Pomacentrus brachialis | Planktivore | Small |
| Pomacentrus coelesti | Grazer | Small |
| Pomacentrus imitator | Grazer | Small |
| Pomacentrus lepidogenys | Planktivore | Small |
| Pomacentrus moluccensis | Spongivore | Small |
| Pomacentrus reidi | Grazer | Small |
| Pomacentrus sp. | Grazer | Small |
| Pomacentrus vaiuli | Spongivore | Small |
| Pomacentrus wardi | Grazer | Small |
| pseudobalistes fuscus | NA | Large |
| Pseudobalistes flavimarginatus | Macro-invertivore | Large |
| Pterocaesio marri | Planktivore | Large |
| Scarus altipinnis | Excavator/scraper | Large |
| Scarus chameleon | Excavator/scraper | Large |
| Scarus dimidiatus | Excavator/scraper | Large |
| Scarus flavipectoralis | Excavator/scraper | Large |
| Scarus frenatus | Excavator/scraper | Large |
| Scarus ghobban | Excavator/scraper | Large |
| Scarus globiceps | Excavator/scraper | Large |
| Scarus niger | Excavator/scraper | Large |
| Scarus oviceps | Excavator/scraper | Large |
| Scarus rivulatus | Excavator/scraper | Large |
| Scarus schlegeli | Excavator/scraper | Large |
| Scarus sp | Excavator/scraper | Large |
| Scolopsis bilineatus | Micro-invertivore | Large |
| Scolopsis monogramma | Micro-invertivore | Large |
| Siganus argenteus | Grazer | Large |
| Siganus corallinus | Grazer | Large |
| Siganus doliatus | Grazer | Large |
| Siganus puellus | Grazer | Large |
| Siganus vulpinus | Grazer | Large |
| Stegastes apicalis | Grazer | Large |
| Stegastes nigricans | Grazer | Large |
| Stethojulis strigiventer | Micro-invertivore | Large |
| Sufflamen chrysopterus | Micro-invertivore | Large |
| Thalassoma hardwicke | Micro-invertivore | Large |
| Thalassoma jansenii | Micro-invertivore | Large |
| Thalassoma lunare | Micro-invertivore | Large |
| Valenciennea strigata | Detritivore | Large |
| Zanclus cornutus | Micro-invertivore | Large |
| Zebrasoma scopas | Grazer | Large |
| Zebrasoma veliferum | Grazer | Large |
